# Immunological Profile of HTLV-1-Infected Patients Associated with Infectious or Autoimmune Dermatological Disorders

**DOI:** 10.1371/journal.pntd.0002328

**Published:** 2013-07-25

**Authors:** Jordana Grazziela Alves Coelho-dos-Reis, Livia Passos, Mariana Costa Duarte, Marcelo Grossi Araújo, Ana Carolina Campi-Azevedo, Andréa Teixeira-Carvalho, Vanessa Peruhype-Magalhães, Bruno Caetano Trindade, Raquel dos Santos Dias, Marina Lobato Martins, Anna Barbara de Freitas Carneiro-Proietti, Antônio Carlos Guedes, Denise Utsch Gonçalves, Olindo Assis Martins-Filho

**Affiliations:** 1 Laboratório de Biomarcadores de Diagnóstico e Monitoração, Instituto René Rachou, FIOCRUZ, Belo Horizonte, Minas Gerais, Brazil; 2 Aaron Diamond AIDS Research Center, Rockefeller University, New York, New York, United States of America; 3 Interdisciplinary HTLV Research Group – GIPH – HEMOMINAS, Belo Horizonte, Minas Gerais, Brazil; 4 Faculty of Medicine, Federal University of Minas Gerais, UFMG, Belo Horizonte, Minas Gerais, Brazil; George Mason University, United States of America

## Abstract

In the present study, the frequency, the activation and the cytokine and chemokine profile of HTLV-1 carriers with or without dermatological lesions were thoroughly described and compared. The results indicated that HTLV-1-infected patients with dermatological lesions have distinct frequency and activation status when compared to asymptomatic carriers. Alterations in the CD4^+^HLA-DR^+^, CD8^+^ T cell, macrophage-like and NKT subsets as well as in the serum chemokines CCL5, CXCL8, CXCL9 and CXCL10 were observed in the HTLV-1-infected group with skin lesions. Additionally, HTLV-1 carriers with dermatological skin lesions showed more frequently high proviral load as compared to asymptomatic carriers. The elevated proviral load in HTLV-1 patients with infectious skin lesions correlated significantly with TNF-α/IL-10 ratio, while the same significant correlation was found for the IL-12/IL-10 ratio and the high proviral load in HTLV-1-infected patients with autoimmune skin lesions. All in all, these results suggest a distinct and unique immunological profile in the peripheral blood of HTLV-1-infected patients with skin disorders, and the different nature of skin lesion observed in these patients may be an outcome of a distinct unbalance of the systemic inflammatory response upon HTLV-1 infection.

## Author Summary

In the present study, the immunological profiles of HTLV-1 carriers with or without dermatological lesions were thoroughly described and compared. The results indicated that HTLV-1-infected patients with dermatological lesions have distinct frequency and activation status than asymptomatic carriers. Alterations in cells and molecules that are important for immune cell function were observed in the HTLV-1-infected group with skin lesions. Additionally, HTLV-1 carriers with dermatological skin lesions have elevated frequency of high proviral load as compared to asymptomatic carriers, which indicates that the virus may be present in higher frequency in those patients. Patients with different skin lesions, autoimmune or infectious, also demonstrated differences in their immunological profile. All in all, these results suggest a distinct and unique immunological profile in the blood of HTLV-1-infected patients with skin disorders, and the different nature of skin lesion observed in these patients may be an outcome of a distinct unbalance of the systemic inflammatory response upon HTLV-1 infection.

## Introduction

Human T-lymphotropic virus 1 (HTLV-1) was the first retrovirus discovered with human clinical importance [1]. While the majority of patients infected with HTLV-1 may remain asymptomatic (95%), some patients (0.2–5%) can develop severe clinical symptoms [2], [3]. In certain endemic regions, the prevalence and incidence of these clinical symptoms among persons infected with HTLV-1 is significantly elevated [4]. Among the many disorders that HTLV-1 carriers may develop, two are paramount: the adult T-cell leukemia/lymphoma (ATL) and a chronic neurological disease, the HTLV-1-associated myelopathy/tropical spastic paraparesis (HAM/TSP).

Contrasting with HIV, HTLV-1 has poor infectivity of CD4^+^ T cells in vitro and the viral particles are scarce in the peripheral blood of HTLV-1 carriers. In spite of that, HTLV-1 proviral DNA is detected particularly in CD4^+^ T cells of the host and the virus is able to induce strong activation and proliferation of many subsets of cells, which allow this retrovirus, together with other factors, to persist by clonal expansion of CD4^+^ T cells of the infected host [5], [6], [7]. There are clear evidences that the immunological profile of HAM/TSP patients is composed by a robust hyperimmune response [8], contrasting with ATL patients. Increased levels of Type-1 cytokines such as IFN-γ, TNF-α, IL-1β, IL-2, IL-6, IL-9 and IL-13 found in HAM/TSP patients are examples of such evidences [9], [10], [11], [12]. Asymptomatic HTLV-1 carriers, however, seem to present a balanced hyperimmune response characterized by altered frequency of proinflammatory IL-12-positive neutrophils and TNF-α-positive monocytes, which is modulated by high frequency of IL-10-secreted by CD4^+^ and CD8^+^ T cells according to Brito-Melo and collaborators (2007) [13].

There is still many unresolved questions on the context of the immunological status of HTLV-1 carriers who develop inflammatory symptoms, but there is no doubt that HAM/TSP is evidently a clinically and immunologically distinct entity that should be considered as unique among other inflammatory disorders associated with HTLV-1. The dermatological disorders associated with HTLV-1, however, have been poorly investigated, which poses a challenge on the management – prognosis and treatment - of patients with such disorders.

Contrasting with the lack of scientific findings on the mechanisms that lead to dermatological disorders in HTLV-1 carriers, infectious and autoimmune dermatitis have been increasingly reported on those subjects, especially in endemic areas [14], [15], [16], [17], [18], [19]. Regarding the immunological context of dermatological disorders in HTLV-1-infected individuals, Nascimento and collaborators (2009) [20] reported that infective dermatitis has similar immunological features to HAM/TSP and could be considered as a risk factor for development of myelopathy.

In face of the elevated prevalence of dermatological disorders among HTLV-1-infected individuals and the scarce knowledge of their immunological status, this study aimed at evaluating frequency, activation and the cytokine and chemokine profile of HTLV-1 carriers with or without dermatological diseases.

## Materials and Methods

### Study population and clinical dermatological evaluation

#### Ethics statement

Informed written consent for laboratory tests was obtained from each and all participants. The HEMOMINAS Foundation, the René Rachou Institute and Federal University of Minas Gerais (number 188/06) Ethics Committees approved the research. This study fulfilled recommendations 196/1996 from the Brazilian National Health Council for research involving humans.

A full description of the patients included in this study is given in Tables 1 and 2. The study population was comprised of 148 persons infected or not with HTLV-1 that have been followed up by the Interdisciplinary HTLV Research Group (GIPH). All these subjects presented negative results for relevant blood-borne pathogens. Use of corticosteroids or other immunosuppressive chemotherapy was an exclusion criterion prior to blood collection. All samples were tested for anti-HTLV-1/2 serology by Enzyme Immunoassay (Ortho Clinical Diagnostics, Rochester, New York, USA), and the samples that were positive for HTLV-1, were confirmed by Western blot (GLD HTLV BLOT 2.4, Genelabs Diagnostics, Singapore). The groups with dermatological disorders (control and HTLV-1+) were categorized by their type of skin lesions that were: infectious skin lesions and autoimmune skin lesions. Autoimmune skin disorders were comprised of xerosis, acquired ichthyosis, psoriasis and pemphigus. Dermatologic diagnoses was performed as described [21].

**Table 1:** Description of patients included in the cell subset analysis.

Cell subset analysis	Abbreviation	Size	Age (yrs)#	Gender M/F*	Dermatological description
Negative Control without lesion	CT L^(−)^	10	35	6/4	None
Negative Control with lesion	CT L^(+)^	18	40	16/2	Skin lesions
Total Negative control	CT	28	38	32/6	
HTLV-1 carriers without lesion	HTLV-1+ L^(−)^	7	39	1/6	None
HTLV-1 carriers with lesion	HTLV-1+ L^(+)^	20	47	9/11	Skin lesions
Total HTLV-1+	HTLV-1+	27	43	10/17	
Negative control with Autoimmune lesions	CT L^(AI)^	8	40	7/1	xerosis, acquired ichthyosis, psoriasis and pemphigus
HTLV-1 carriers with Autoimmune lesions	HTLV-1+ L^(AI)^	9	44	4/5	
Negative control with Infectious lesions	CT L^(INF)^	10	42	9/1	dermatophytosis, candidiasis and *Malassezia* infections
HTLV-1 carriers with Infectious lesions	HTLV-1+ L^(INF)^	11	49	5/6	

# - Age average in years;

* M/F – Male/Female ratio.

**Table 2:** Description of patients included in the cytokine/chemokine analysis.

Cytokine/chemokine profile analysis	Abbreviation	Size	Age (yrs)#	Gender M/F*	Dermatological description
Negative Control without lesion	CT L^(−)^	19	47	10/9	None
Negative Control with lesion	CT L^(+)^	25	41	14/11	Skin lesions
Total Negative control	CT	44	44	24/20	
HTLV-1 carriers without lesion	HTLV-1+ L^(−)^	16	38	3/13	None
HTLV-1 carriers with lesion	HTLV-1+ L^(+)^	33	48	15/18	Skin lesions
Total HTLV-1+	HTLV-1+	49	43	18/31	
Negative control with Autoimmune lesions	CT L^(AI)^	11	45	6/5	xerosis, acquired ichthyosis, psoriasis and pemphigus
HTLV-1 carriers with Autoimmune lesions	HTLV-1+ L^(AI)^	14	47	6/8	
Negative control with Infectious lesions	CT L^(INF)^	14	37	8/6	dermatophytosis, candidiasis and *Malassezia* infections
HTLV-1 carriers with Infectious lesions	HTLV-1+ L^(INF)^	19	49	9/10	

# - Age average in years;

* M/F – Male/Female ratio.

### Flow cytometric immunostaining

For FACS immunostaining, antibodies including: anti-human CD3-FITC, anti-human CD4-FITC, anti-human CD8-FITC, anti-CD14-FITC, anti-human CD16-PE, anti-human CD19-FITC, anti-human CD56-FITC, and anti-human HLA-DR-PE (Pharmingen, San Diego, CA, USA) were utilized. Whole blood staining of individual samples was performed according to manufacturer's instructions and adapted as described [22].

For acquisition and analysis, identification of the subsets was performed by the dual-color immunophenotyping method within the lymphocyte or monocyte scatter gate. All results were expressed as percentage of positive cells for the different subsets of cells analyzed in this study.

### CBA

To assess the levels of the cytokines – TNF-α, IL-12, IL-10, IL-6 and IL-1β – and chemokines – CCL2 (MCP-1), CCL5 (RANTES), CXCL8 (IL-8), CXCL9 (MIG), CXCL10 (IP-10) – in the sera from HTLV-1 carriers and controls, Cytometric Bead Array kits (BD Biosciences, California, USA) were utilized according to manufacturer's protocol and adapted as described [23]. Analysis of raw data was performed using the FlowJo cytometry analysis software (FlowJo, Stanford, USA) and the median fluorescence intensity (MFI) of each bead cluster was evaluated to calculate the cytokine concentration in the sera of patients. Cytokines concentrations were extrapolated according to the standard curve created by serial dilutions of the positive control. The concentrations of cytokines were expressed in pg/mL.

### Quantitation of HTLV-1 proviral load

To quantify the HTLV-1 proviral load of the HTLV-1 seropositive individuals, peripheral blood was collected from the patients in tubes containing EDTA anticoagulant. DNA was isolated from peripheral blood by column extraction (QIAamp DNA Blood kit; Qiagen GmbH,Hilden, Germany) and HTLV-1 proviral load was quantified by a real-time SYBR Green PCR method as previously described [24]. The value for the HTLV-1 proviral load was reported as [(pol average copy number)/(albumin average copy number/2)]×10^4^ and expressed as the number of HTLV-1 copies/10^4^ cells.

### Statistical analysis

The ANOVA one-way with Dunnet's post-test was utilized to compare the groups for all the immunological parameters evaluated. Pearson's and Spearman's correlation tests were utilized to compare cytokine levels with HTLV-1 proviral load of the patients. The Prism GraphPad Software version 5.0 (San Diego, CA, USA) was applied for the statistical analysis, and differences between groups with *P* values <0.05 were considered as statistically significant and indicated in the figures as letters or a line connecting the two different groups.

## Results

### Nature of skin lesion — autoimmune or infectious — seems to influence in the immunophenotypic profile of HTLV-1 carriers with dermatological disorders

**Figure 1:**
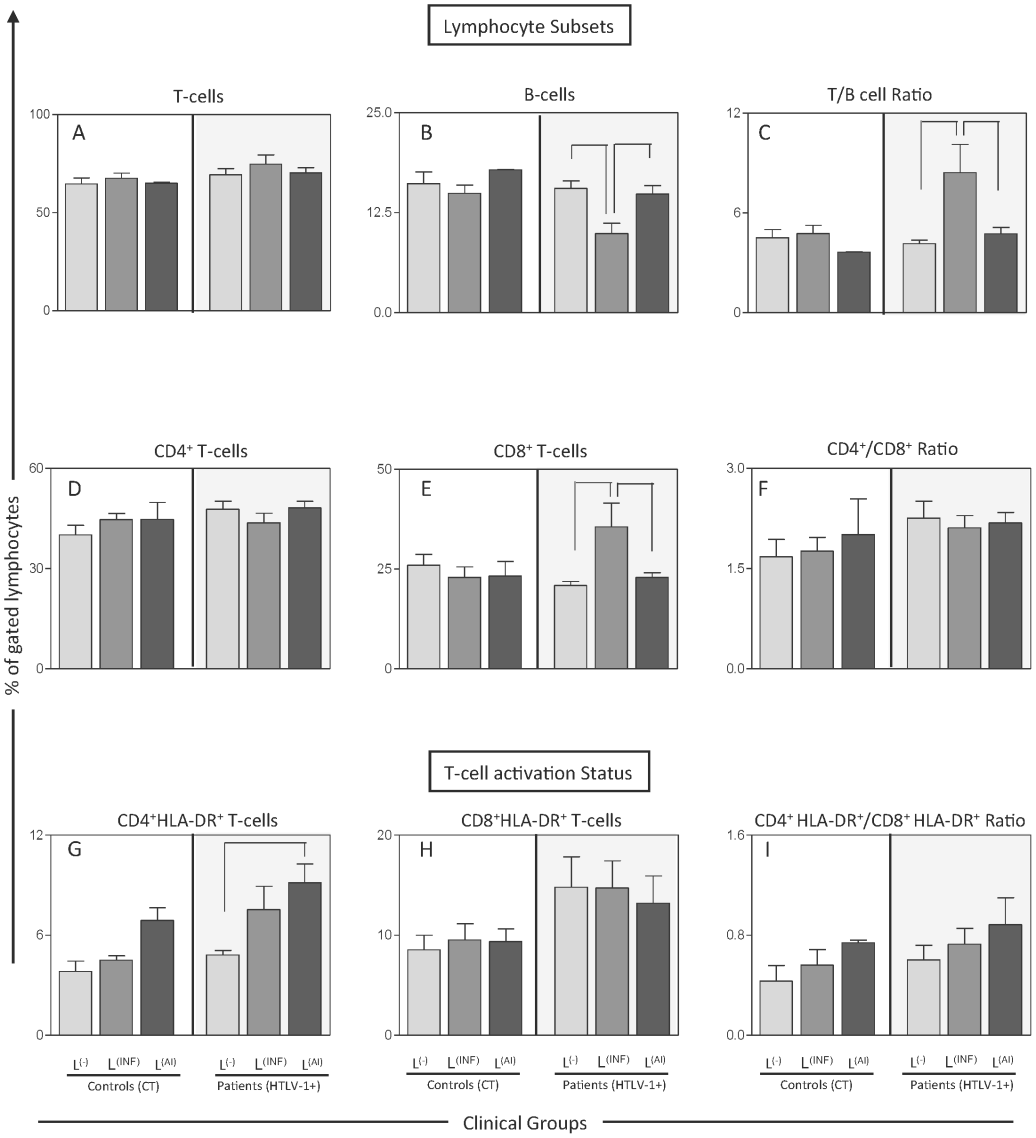
Phenotypic profile of HTLV-1-infected patients and uninfected controls with skin lesions. Phenotypic studies were performed by a FACS double-labeling protocol as described in materials and methods. The results are expressed as mean percentage (%) ± standard error of (A) CD3^+^ T cells, (B) CD19^+^ B cells, (C) T/B ratio, (D) CD4^+^ T cells, (E) CD8^+^ T cells, (F) CD4^+^/CD8^+^ ratio, (G) CD4^+^HLA-DR^+^ (H) CD8^+^HLA-DR^+^ (I) CD4^+^HLA-DR^+^/CD8^+^HLA-DR^+^ in the peripheral blood from HTLV-1-infected patients and controls with infectious (L^(INF)^ = dark gray rectangles) and autoimmune (L^(AI)^ = black-gray rectangles) skin lesions as well as carriers and controls without skin lesions (L^(−)^ = light gray rectangles). Significant differences at *P*<0.05 are expressed by connecting lines.

Figure 1 shows the frequency of the subset of cells evaluated in HTLV-1 carriers with skin lesions. The data demonstrated that the two types of skin lesions present different immunophenotypic profiles. The HTLV-1-infected group with infectious dermatological lesions presented statistically decreased frequency of B cells, increased frequency of CD8^+^ T cells, and increased T/B cell ratio when compared to the HTLV-1-infected group with autoimmune skin lesions. The HTLV-1-infected group with autoimmune skin lesions showed increased levels of CD4^+^ HLA-DR^+^ T cells when compared to the HTLV-1-infected group without lesions.

### Macrophage-like and NKT cell subsets are increased in HTLV-1 carriers with skin lesions

Regarding the innate immune response of HTLV-1 carriers with skin disorders, Table 3 shows the frequency of the innate and regulatory cell subsets evaluated. Macrophage-like (CD14^+^CD16^+^/CD14^+^), pro-inflammatory monocytes (CD14^+^CD16^+^HLA-DR^++^/CD14^+^CD16^+^), NK cells (CD3^−^CD16^+/−^/CD56^+/−^/CD3^−^), NKT cells (CD3^+^CD16^+/−^/CD56^+/−^/CD3^+^) and regulatory T cells (CD4^+^CD25^+^^High^) were analyzed in the peripheral blood of HTLV-1 carriers with and without lesions as well as control (Table 3). The results demonstrated a statistically significant increase in the macrophage-like subset in the HTLV-1-infected group with skin lesions when compare to uninfected control with skin lesions, while the proinflammatory monocytes were decreased in the HTLV-1-infected group with and without skin lesions when compared to their respective controls. The NK subset showed increased percentage in the HTLV-1-infected group with and without skin lesions when compared to their respective controls. Interestingly, the NKT subset showed statistically significant increase in the HTLV-1-infected group with skin lesions when compared to the uninfected controls with skin lesions. The same difference was not observed in the HTLV-1-infected group without skin lesions when compared to its respective control.

**Table 3:** Phenotypic profile of innate immunity subsets.

Cell Phenotypes*	Clinical Groups					
	Control			HTLV-1^+^		
	All	L^(−)^	L^(+)^	All	L^(−)^	L^(+)^
**CD14^+^ CD16^+^/CD14^+^**	8.5±2.2	8.8±2.7	8.1±1.6	**12.3±5.7^a^**	11.3±5.0	**12.8±6.1^c^**
**CD14^+^CD16^+^HLADR^++^/CD14^+^CD16^+^**	65.5±13.7	65.4±12.6	65.7±15.4	**50.5±15.9^a^**	**49.5±13.1^b^**	**50.9±17.4^c^**
**CD3^−^CD16^−/+^CD56^−/+^/CD3^−^**	10.7±4.1	11.5±4.6	10.0±3.8	**18.2±8.3^a^**	**18.6±6.0^b^**	**18.1±19.2^c^**
**CD3^+^CD16^−/+^CD56^−/+^/CD3^+^**	0.5±0.3	0.6±0.2	0.4±2.1	**0.9±0.7^a^**	0.6±0.2	**1.0±0.2^c^**
**CD4^+^CD25^HIGH^**	1.9±1.3	2.2±1.7	1.7±1.1	1.9±1.2	1.9±1.0	1.9±1.3

Monocyte subsets, NK-cells and NKT-cells and Regulatory T-cells in the peripheral blood of HTLV-1-infected patients and uninfected controls according to the absence (L^(−)^) or presence (L^(+)^) of dermatological Lesions.

* Data are expressed as mean percentage (%) ± standard error of cell phenotype within a selected gated cell subset shown after the slash, except for CD3^−^CD16^−/+^CD56^−/+^ and CD4^+^CD25^HIGH^ that were analyzed within gated lymphocytes.

Significant differences at *P*<0.05 are highlighted by letter “a”, “b” and “c” for difference between pairs of subgroups “All”, L^(−)^ and L^(+)^, respectively.

### Unique cytokine and chemokine profile of HTLV-1 carriers with dermatological lesions

**Figure 2:**
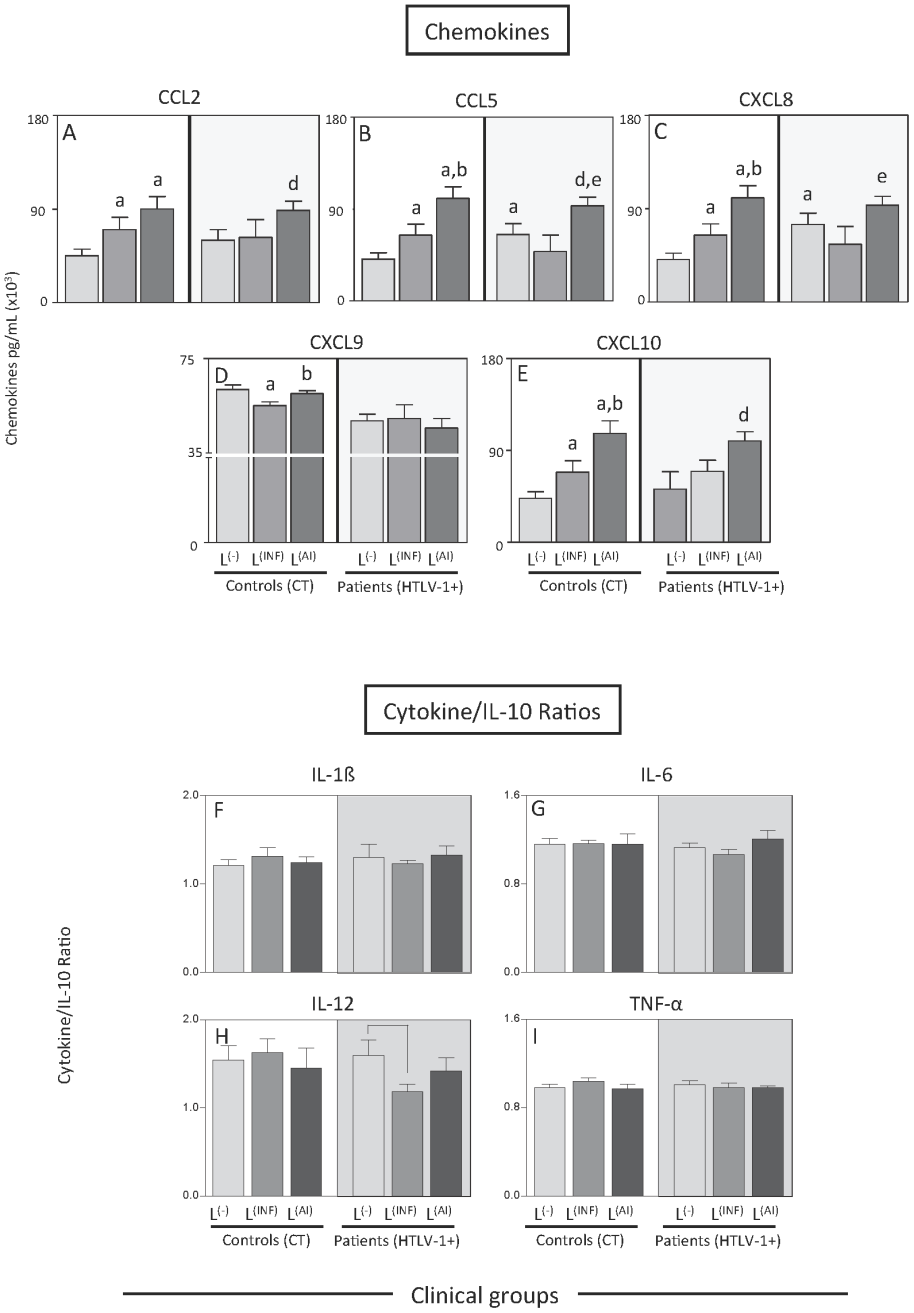
Plasmatic levels of chemokines and cytokine ratios among HTLV-1-infected patients with skin lesions. Serum chemokines and cytokines quantitation were performed by CBA as described in materials and methods. The results are expressed as concentration in pg/mL (×10^3^) ± standard error of (A) CCL2, (B) CCL5, (C) CXCL8, (D) CXCL9, (E) CXCL10 in the serum of HTLV-infected patients and controls with infectious (L^(INF)^ = dark gray rectangles) and autoimmune (L^(AI)^ = black-gray rectangles) skin lesions as well as carriers and controls without skin lesions (L^(−)^ = light gray rectangles). Significant differences at *P*<0.05 are highlighted by letters for difference of a group as compared to control L^(−)^ indicated by “a”, to control L^(INF)^ indicated by “b”, to control L^(AI)^ indicated by “c”, to HTLV-1+ L^(−)^ indicated by “d” and to HTLV-1+ L^(INF)^ indicated by “e”. The ratio between cytokines and IL-10 for all groups was calculated for (F) IL-1β, (G) IL-6, (H) IL-12, (I) TNF-α. Significant differences are highlighted by connecting lines.

To verify whether the nature of skin lesion was associated with altered chemokine and cytokine levels, the levels of these molecules in the infectious skin lesions group and the autoimmune skin lesions group were evaluated and contrasted (figure 2). The chemokines CCL5 and CXCL8 are significantly increased in the HTLV-1-infected group with autoimmune dermatological lesions when compared to the HTLV-1-infected group with infectious skin lesions. The chemokine CXCL10 was statistically increased in the HTLV-1-infected group with autoimmune skin lesions when compared to HTLV-1-infected group without lesions. The cytokine ratio analysis showed a decrease in the IL-12/IL-10 ratio in the HTLV-1-infected group with infectious dermatological lesions when compared to the HTLV-1-infected group without skin lesions, which indicates that the decrease in this ratio observed previously is attributed to the group with infectious skin lesions.

**Figure 3:**
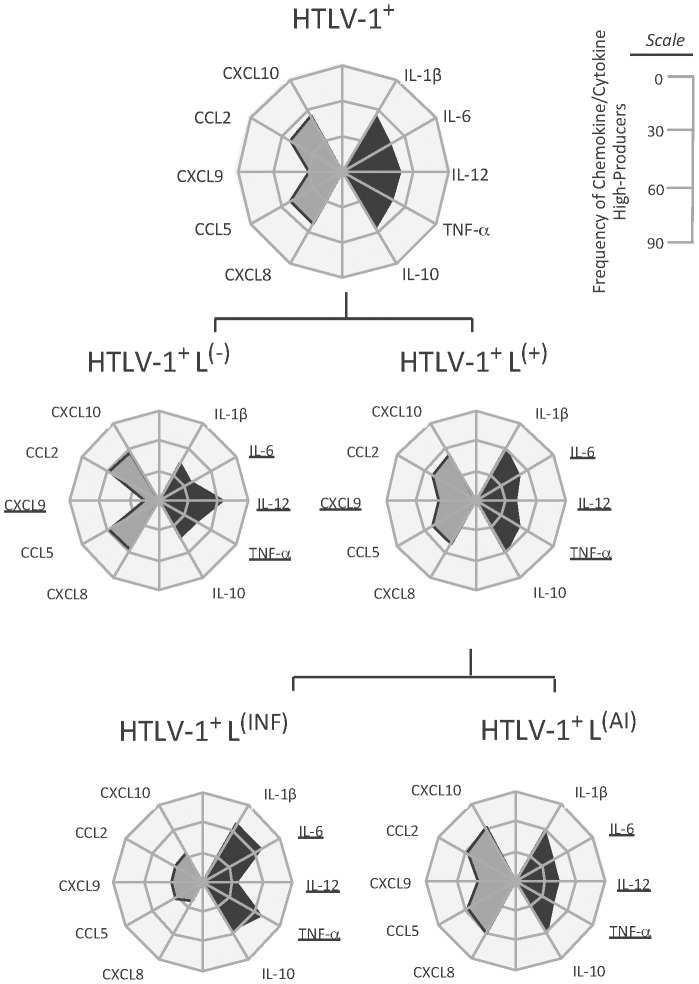
Frequency of high producers of chemokines and cytokines among HTLV-1-infected patients with skin lesions. The group with skin lesions was subdivided in the sub-groups of patients with infectious and autoimmune skin lesions. The frequency of high producers of chemokines (in gray) and cytokines (in black) is displayed in radar graphs with three stages (30% each) from 0 to 90% (as described in the scale located in the upper right corner of the graph) and it was calculated using the global arithmetic mean percentage of chemokines/cytokine levels as a threshold to define high and low producers.

Figure 3 shows the panoramic chemokine and cytokine profile of high producers from the HTLV-1-infected and control groups displayed in radar graphs. The altered chemokine and cytokine profile identified before was sustained when the high producers from each group were compared. Remarkably, CXCL9 shows evident decrease associated to HTLV-1 infection. The high producers of the HTLV-1-infected group with infectious skin lesions demonstrate a clear and statistically significant increase of TNF-α and IL-6 when compared to the HTLV-1-infected group with autoimmune skin lesions. This group also shows a significant decrease in IL-12 when compared to the HTLV-1-infected group without lesions. Radar graphs with cytokine profile of high producers from the Control groups are displayed in Figure S1.

### High levels of proviral load are more frequent in HTLV-1 carriers with dermatological skin lesions

**Figure 4:**
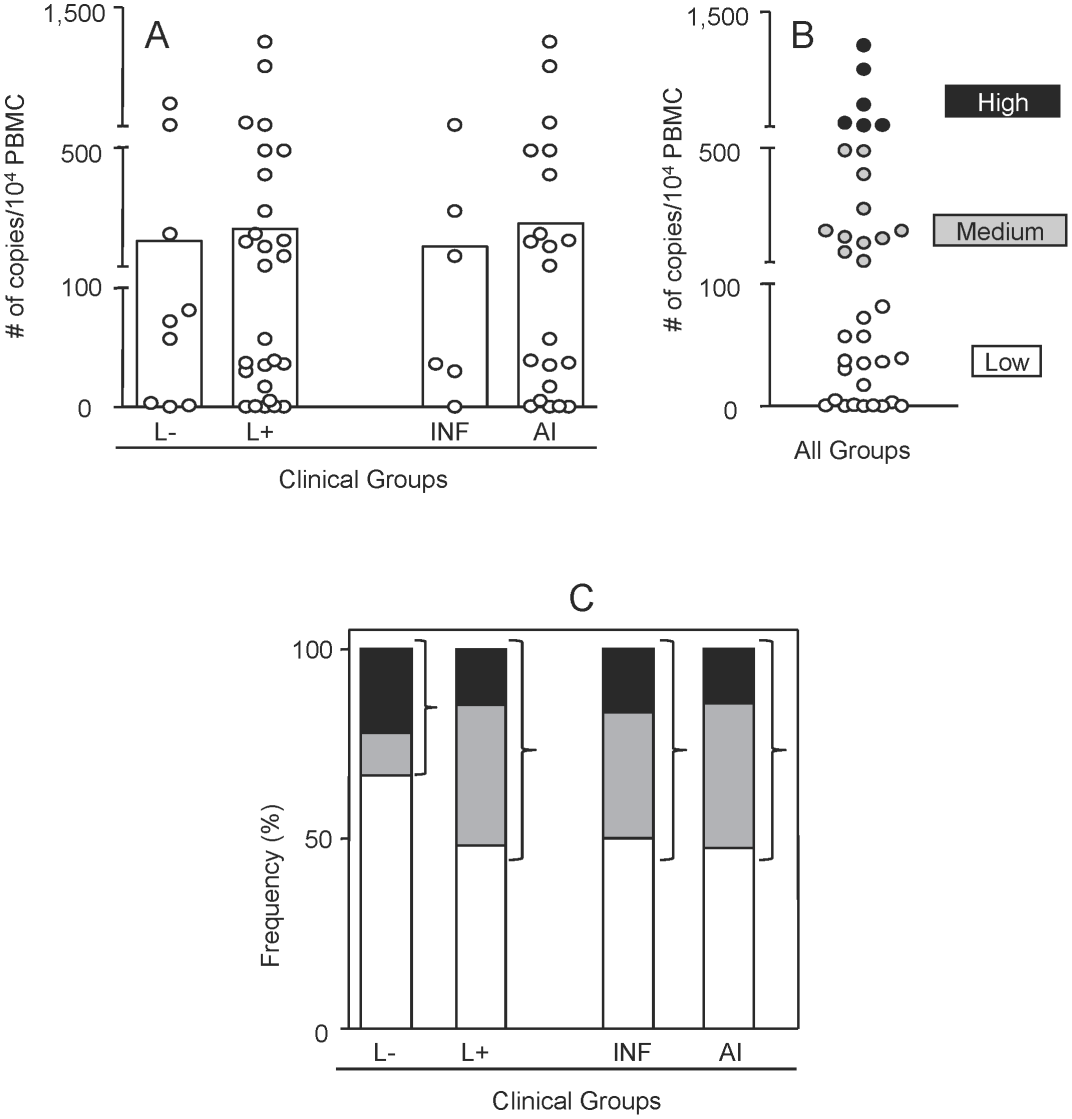
Proviral load of HTLV-1-infected patients without and with infectious or autoimmune skin lesions. (A) Proviral load was measured by Real Time PCR as described in Materials and Methods and results are expressed in number of HTLV-1 genome copies per 10^4^ peripheral blood mononuclear cells (PBMCs). The frequency of high (black rectangles #6 – 2 without skin lesions and 4 with skin lesions) medium (gray rectangles #11 – 1 without skin lesions and 10 with skin lesions) and low (white rectangles #19 – 6 without skin lesions and 13 with skin lesions) copy number was defined by the global arithmetic mean of all data (B) and individual groups (C).

The proviral load of peripheral blood mononuclear cells from HTLV-1-infected patients with or without skin lesions was evaluated. Figure 4 shows that the mean of proviral load in the groups with and without skin lesions or in the groups with infectious or autoimmune skin lesions did not differ statistically (figure 4). On a different analysis, these groups were merged and the patients were classified as possessing: high (≥1000 proviral copies/10^4^ cells), medium (>100 and <1000 proviral copies/10^4^ cells) or low proviral load (≤100 proviral copies/10^4^ cells). The frequency of patients with high, medium or low proviral load from each group demonstrated that HTLV-1 carriers without skin lesion contained the lowest percentage of patients with high-medium proviral load and the highest percentage of patients with low proviral load.

### Proinflammatory and regulatory cytokine balance in the peripheral blood of HTLV-1 carriers with different dermatological skin lesions correlates with the proviral load

**Figure 5:**
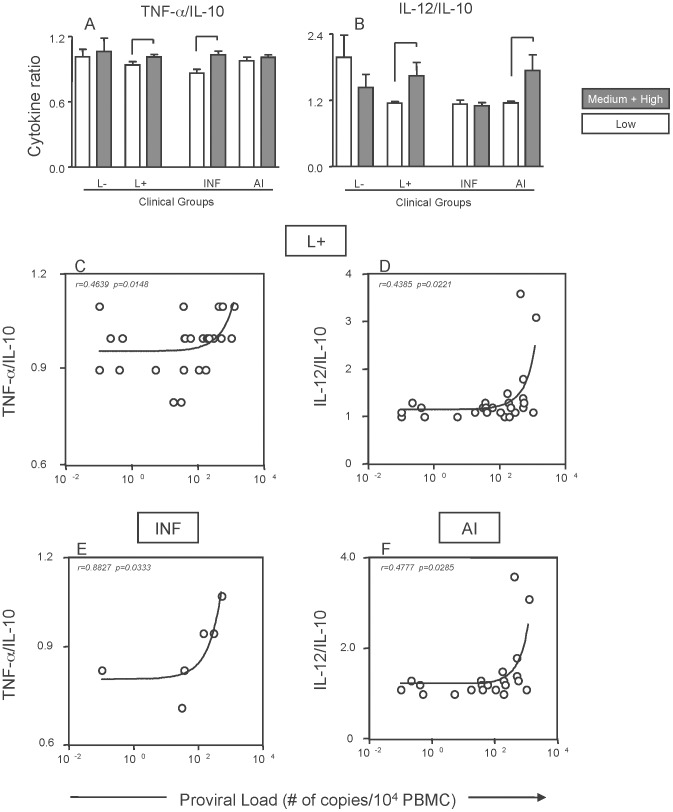
Cytokine balance versus proviral load among HTLV-1-infected patients with infectious and autoimmune skin lesions. Ratio of TNF-α/IL-10 (A) and IL-12/IL-10 (B) of patients with high and medium proviral load (gray rectangles) as compared to patients with low proviral load (white rectangles). Spearman's correlation was calculated for proviral load and TNF-α/IL-10 (C) and IL-12/IL-10 (D) of HTLV-1-infected patients with skin lesions. Spearman's correlations for TNF-α/IL-10 (E) and IL-12/IL-10 (F) were also calculated for the sub-groups of HTLV-1-infected patients with skin lesions of infectious or autoimmune nature, respectively. Statistically significant correlations at *P*<0.05 are displayed in the graphs along with spearman's coefficient (r).

To evaluate whether the immunological features screened in the present study correlated with presence of the HTLV-1 provirus, the chemokines and cytokines levels were compared among patients of each group subdivided previously as high-medium and low proviral load carriers (figure 5). Among all the chemokines and cytokines/IL-10 ratio tested, TNF-α/IL-10 and IL-12/IL-10 ratios were statistically higher within HTLV-1-infected patients with infectious and autoimmune skin lesions, respectively, bearing high-medium proviral load. Spearman correlation's analysis confirmed significant correlation between proviral load and TNF-α/IL-10 ratio (r = 0.8827; p = 0.0333) in patients with infectious skin lesions, and proviral load and IL-12/IL-10 ratio (r = 0.4777 p = 0.0285) in patients with autoimmune skin lesions.

## Discussion

Dermatological alterations are quite common in HTLV-1-carriers. Several reports have described dermatological diseases such as infective dermatitis as a prodromic manifestation of HAM/TSP and ATLL progression [14], [19], [25], [26], [27]. Although these cutaneous disorders are common in HTLV-1 carriers, it is still unclear how HTLV-1 and its machinery could induce dermatological lesions. The HTLV-1 genome in skin biopsies may be difficult to detect even by highly sensitive methods [28], [29], [30], which could be partially explained by the evidence of deleted HTLV provirus in the cutaneous lesions of mycosis fungoides patients [29], [31], [32].

However, the association between HTLV and Mycosis fungoides has remained controversial since few patients with mycosis fungoides are seropositive for antibodies to structural components of HTLV 1 and 2 virions and not all of the patients present evidence of *tax* sequences [33]. Other studies support this controversy but challenge the findings of HTLV proviral sequences in patients with Mycosis fungoides after evaluation of these patients's samples by the same molecular probes and techniques utilized in previous reports. These studies have indicated a possible misdiagnosis of Mycosis fungoides or little association with HTLV provirus [34], [35], [36], [37], [38]. These findings suggest that the association of HTLV provirus and Mycosis fungoides is still unclear and should be considered with care. In addition, the tissue damage may not be caused by a direct effect of the virus, but actually originated by the systemic and local deregulation of the immune response in cutaneous disorders induced by the HTLV-1.

**Figure 6:**
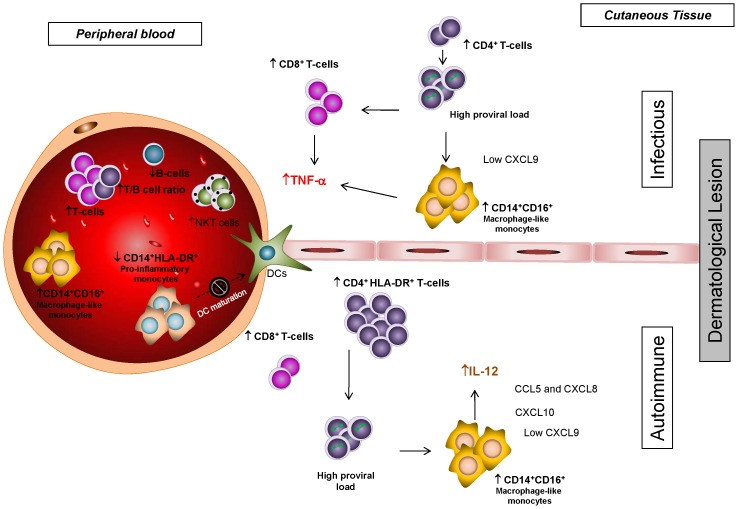
Immunoregulation in HTLV-1-infected patients with skin lesions of infectious or autoimmune nature. The schematic representation illustrates the cytokines, chemokines and cell populations altered in the peripheral blood of patients chronically infected by HTLV-1 with infectious or autoimmune skin lesions. These immunological features give insight to the understanding of how the immune systemic biomarkers may be involved in the generation of protective and pathogenic responses and their potential association with proviral load and skin lesion.

Brito-Melo (2002 and 2006) and Coelho-dos-Reis (2007) and collaborators proposed that, among the many altered subset of cells on HTLV-1 carriers from Brazil, the decreased frequency of B cells, the increased frequency of activated CD8^+^ T cells and the T/B cell ratio are characteristic of HAM/TSP patients [13], [22], [39]. In the present study, these immunological markers together with others were evaluated in HTLV-1 carriers with skin lesions. The results indicated that HTLV-1-infected carriers with dermatological disorders also display alterations similar to the ones found in HAM/TSP patients from Brazil. However, HTLV-1-infected patients with skin lesions showed other immunological features that suggest a distinct and unique immunological profile in their peripheral blood. Figure 6 illustrates a schematic representation of these unique immunological profiles in the peripheral blood of HTLV-1-infected patients with infectious or autoimmune skin disorders and how these profiles could be associated with each particular skin lesion.

In the present study, it was identified that patients with infectious dermatological lesions had similar frequency and activation status to HAM/TSP patients previously described, however, the chemokine profile seems to be divergent considering previous findings. Previous studies have demonstrated that levels of serum chemokines discriminate clinical HAM/TSP disease from HTLV-1 carrier state [40]. Increased serum levels of CXCL10 was previously observed in HAM/TSP patients when compared to asymptomatic carriers from a Brazilian cohort of HTLV-1-infected patients [40], which converges with the results of the present study that also showed an increase of CXCL10 in HTLV-1-infected patients with autoimmune skin lesions. In the present study, a decrease in CXCL9 was also found in HTLV-1-infected patients with skin lesions regardless of the lesion type when compared to uninfected controls. Contrasting with this finding, high levels of CXCL9 were found to be features of patients with HAM/TSP when compared to asymptomatic carriers [40]. HTLV-1 patients with autoimmune skin lesions presented elevated CCL5 and CXCL8. In vitro evidence of HTLV-1 induction of chemokine secretion were reported showing that patients as well as cell lines infected with HTLV-1 express chemokines such as CCL2, CCL3, CCL4, CCL5, CXCL8, CXCL10 and CCL22 [41], [42],[43],[44],[45] and some of these chemokines may be induced by the transactivator Tax protein [41], [45]. Many types of cells are able to produce chemokines and their levels are variable, even in healthy subjects, therefore future studies performed in vitro and ex vivo should be carried out to understand whether HTLV-1 patients with different dermatological lesions indeed have a distinct chemokine secretion profile and also comparing the chemokine profile of HAM/TSP specially regarding their serum levels of CXCL9.

The monocytic populations seem to be altered in HTLV-1 patients with skin lesions. The macrophage-like monocytes (CD14^+^CD16^+^/CD14^+^) were increased in the HTLV-1 group with skin lesions while the proinflammatory monocytes (CD14^+^CD16^+^HLA-DR^++^/CD14^+^CD16^+^) were decreased in both HTLV-1-infected groups with and without skin lesions. These results are in agreement with previous results that showed downregulation of CD14 exerted by HTLV-1 infection and impairment of differentiation of monocytes into macrophages or dendritic cells from HTLV-1-infected patients [46], [47]. This unbalance of the monocytic subsets in the peripheral blood could ultimately affect migration and homing of monocyte-derived subsets, such as dendritic cells in the tissues, which may breach the skin defenses and allowing for lesion formation in HTLV-1 infected patients [47].

On the proviral load analysis, considering the low number of the patients with high proviral load (#6 – 2 without skin lesions and 4 with skin lesions), we merged the patients with high and medium proviral load to allow for a better understanding of the association of proviral load and skin lesion. In this analysis, the group with skin lesions presented higher frequency of patients with the higher-medium proviral load (14/27) as compared to the group without lesion (3/9).

The balance of cytokines also demonstrated to be uniquely altered in HTLV-1 patients with skin lesions. The association of elevated proviral load and exacerbated immune response namely by increase of CD8^+^ T cell frequency and cytokines such as IFN-γ and TNF-α was comprehensively described in patients with HAM/TSP. However, little is still known about this equilibrium in other HTLV-1-associated diseases. In agreement with previous findings on patients with myelopathy, the elevated proviral load in HTLV-1 patients with infectious skin lesions correlated significantly with TNF-α/IL-10 ratio, while IL-12/IL-10 ratio seems to be the ratio driving the unbalanced cytokine response in patients with autoimmune skin lesions. The reason that determines why the cytokine balance is different in the infectious versus autoimmune skin lesions still remains to be investigated. One hypothesis to explain this difference in cytokine balance could be the presence of different T cell subsets in the peripheral blood of patients with skin lesions. While the HTLV-1-infected group with infectious dermatological lesions presented increased frequency of CD8^+^ T cells, the HTLV-1-infected group with autoimmune skin lesions showed increased levels of CD4^+^ HLA-DR^+^ T cells. These different T cell populations could provide a microenvironment that modulates the production of TNF-α and especially IL-12 from macrophage-like monocytes, which are increased in patients with skin lesions (Figure 6).

These results show that the two types of skin lesions, infectious and autoimmune, seem to be associated with two distinct immunological cytokine balances in patients with higher proviral load. Understanding the different systemic inflammatory environments pictured in the peripheral blood of HTLV-1 carriers with skin lesions may be useful to unveil the systemic and local pathogenesis directly or indirectly induced by HTLV-1 as well as for future application of these immunological biomarkers in clinical investigations.

## Supporting Information

Figure S1 **Frequency of high producers of chemokines and cytokines among controls with and without skin lesions.** The group with skin lesions was subdivided in the sub-groups of patients with infectious and autoimmune skin lesions. The frequency of high producers is displayed in radar graphs for each of the groups and it was calculated using the global arithmetic mean percentage of chemokines/cytokine levels as a threshold to define high and low producers.
(TIF)
