# Supplementary figures and images for: Immunological Profile of HTLV-1-Infected Patients Associated with Infectious or Autoimmune Dermatological Disorders
Source: PLoS Negl Trop Dis. 2013 Jul 25;7(7):e2328. doi: 10.1371/journal.pntd.0002328 (PMC3723575; doi:10.1371/journal.pntd.0002328)

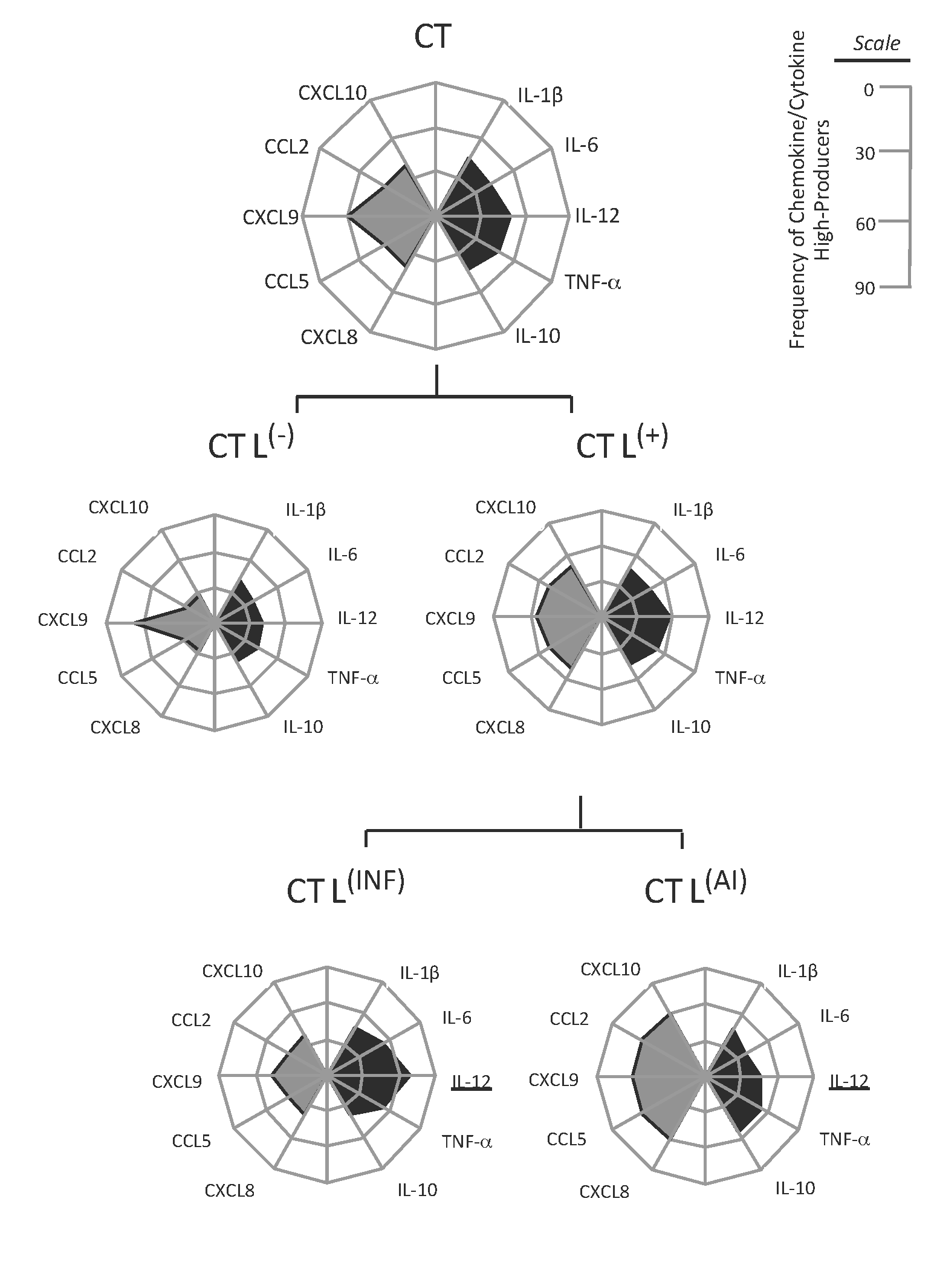

Supplement: Figure S1 — Frequency of high producers of chemokines and cytokines among controls with and without skin lesions. The group with skin lesions was subdivided in the sub-groups of patients with infectious and autoimmune skin lesions. The frequency of high producers is displayed in radar graphs for each of the groups and it was calculated using the global arithmetic mean percentage of chemokines/cytokine levels as a threshold to define high and low producers. (TIF) [file pntd.0002328.s001.tif]
